# Relationship of Leadership and Envy: How to Resolve Workplace Envy with Leadership—A Bibliometric Review Study

**DOI:** 10.3390/jintelligence9030044

**Published:** 2021-09-07

**Authors:** Hongda Liu, Jiejun Geng, Pinbo Yao

**Affiliations:** 1 School of Economics & Management, Tongji University, Shanghai 200092, China; liuhoda@163.com or; 2 School of Management, Shanghai University, Shanghai 200444, China; 3 International School of Business Administration, Shanghai International Studies University, Shanghai 200333, China; hanruishiye2019@163.com

**Keywords:** envy, leadership, bibliometric review, binary relationship, dual-track theoretical model

## Abstract

In recent years, workplace envy has gradually become a hot research topic for organizational behavior. Scholars have explored the antecedents and consequences of envy following the traditional research paradigm. The latest leadership theory also provides new ideas for its development. Although the traditional methods continue to optimize the research on the relationship between leadership and envy, they still do not fully reflect the binary logical relationship between the two and cannot offer sufficient explanatory power for the psychological activities and behaviors of employees and supervisors. In this paper, two pieces of bibliometric software, CiteSpace (American, Drexel University) and Histcite (American, Clarivate Analytics), were used in order to analyze the previous literature in regard to providing a theoretical basis, the main content, and the stages of development. Based on the integration, we propose a dual-track theoretical model of leadership and envy as the prediction framework for future research. The research has returned to the intelligent attribute of leadership and believes that effective leadership can adjust the existence of various types of envy and transform it into the actual productivity of the workplace.

## 1. Introduction

With the increases in social mobility and proximity (Lu et al. 2013), the legitimacy of interests and the fairness of institutions have gradually become important psychological demands of people. The social comparisons brought about by the gaps in benefits and systems trigger people’s emotional evaluations and produce complex emotional states of negative cognition. The rapid development of psychology and interpersonal relationships has resulted in this kind of psychological experience—i.e., envy—attracting widespread attention in academic circles. The research on envy is based on psychology and is enriched by the diversity of the audience, the diversification of subject areas, and the complexity of psychological activities, and it has characteristics that are atypical for psychological research: envy can be managed autonomously, and even can be determined by others and the external environment. Through reasonable guidance and intervention, envy can be used as an “office tool” to shift the dual extremes of continuous emotional variables—the theory of envy is harmful to the theory of beneficial envy. Envy can be destructive or can lead to promotions, and it also has the functional role of guiding the development of people’s hearts and organizations.

At present, many documents have explored all aspects of envy-driven activities, which deeply reflect the occurrence, reaction mechanism, and external behavior of envy (Fu 2010). However, in general, such research still follows the research paradigm of psychology, without considering the combination of envy and the environment and new functions of envy given by the times. In fact, envy appears more in the workplace these days and has a turbulent influence on interpersonal relationships and the atmospheres of offices, e.g., it can promote hatred for power and wealth. This puts higher demands on organizational leadership and mechanisms for the redirection of envy. Analyzed at the macro level, such as national and regional governance, envy is a subjective emotional behavior of the led or controlled subject, triggered by the unbalanced nature of their rights and interests, under the control of coercive power. Even in the game of central and local governments, envy can be significant and lead to regional differences in governance, not to mention the narrow space of psychological confrontation that is the workplace, where the relationship between leadership and envy will be even stronger (Liu et al. 2021). This article integrates the views of the predominant literature and sorts out the research content and developmental stages of envy-linked activities based on a leadership perspective. Herein, we analyzed the lack of existing research, the emotional connotations of envy in the organizational environment, and the internal mechanism that affects interpersonal behavior; additionally, we revealed the key path for effective leadership that uses the positive aspects of envy. We also constructed an overall theoretical framework and put forward the dual-track theoretical hypothesis of envy-linked activities from the perspective of leadership.

Emotional intelligence is the expression of the full range of our emotional expression, including will, character, disposition and emotions, mainly in the handling of human relationships. Envy is an important part of emotional expression and is closely linked to the intelligence of the person expressing the emotion. Envy, admiration and worship of employees are common expressions of emotion in the workplace. As a manager, using your own wisdom and experience to deal with the psychological changes of your staff and to guide them in a positive direction reflects the strength of your leadership. As an employee, the ability to use one’s own wisdom to deal with the ever-changing interpersonal relationships in the face of changes in the workplace, to maintain emotional stability in the face of frustration and ultimately to use one’s own wisdom to resolve difficult situations is the full expression of emotional intelligence in the workplace. In the workplace, there is always a game between managers and employees, and it is the responsibility of managers to use management wisdom and management behavior to manage the relationship between employees and employees, and between employees and leaders.

Based on the above analysis, this article has formed the following research questions: (1) What are the definitions of jealousy and leadership, and where is the intersection of the relationship between the two; (2) What kind of theory is used to form what kind of leadership-envy relation, what kind of realistic or theoretical background this connection originates from; (3) The mechanism of envy itself, including the cause and effect, etc.; (4) Under different forms of envy, what kind of changes and manifestations of leadership are produced; (5) The change in the relationship envy with the leader, and the circumstances under which this change occurred; (6) The path of the leader’s intervention in envy.

To this end, we initially formed the following hypothetical judgment: We believe that envy has two sides, and the formation of its two sides stems from the staff or the working environment. Nevertheless, the two-sided result depends on the role of leadership. Positive and reasonable leadership is a kind of workplace wisdom that will drive human and envy relationships. Furthermore, wrong leadership can worsen goodwill envy. In the same way, positive and correct leadership can also transform malicious envy into well-meaning envy. Therefore, the rational use of the influence of leadership will be the key to regulating workplace emotions and giving play to the “little intelligent” of the workplace.

This paper’s literature review and inherent analysis of envy, leadership, and the workplace (particular environments) allow for the following research objectives:

- From the conceptual level: (1) To further clarify the difference between envy and jealousy, the work environment and workplace climate are clarified to shape the particular concept of envy (Salovey and Rodin 1984). (2) A clear conceptualization of leading and leadership, extending the noun attribute of the concept to the verb state, i.e., an analysis of leadership activities in dynamic behavior and their impact on the work environment (Hupka 1984). (3) To pay particular attention to the workplace, identify its specific properties and heterogeneity with other social environments (Silver and Sabini 1978).
- At the theoretical level, the essential theoretical foundations and empirical thinking on envy, leadership, and the workplace are sorted out and summarized by previous authors, from which the potential relationships between the three are summarized, and the intersection areas or interweaving points of findings are clarified. Ultimately, this paper lays the foundation for the literature synthesis and the organization of ideas utilizing measurement.
- At the relational level, the literature on the relationship between leadership and envy is summarized using bibliometric methods to form a lineage and evolutionary history of literature research. The relationship between leadership and envy in the workplace environment was analyzed in real-life and historical frameworks and different years. It is concluded that the relationship between leadership and envy has evolved from isolation to a potential single influence (envy stemming from the non-benign distribution or role of leadership), to a potential two-way influence (too much workplace envy putting demands on leadership), to a direct control mechanism (through the role of leadership, the transformation of benign envy can be achieved, and the formation of a large amount of positive envy can contribute to rapid corporate development (Leach and Spears 2008)).
- This paper proposes a two-track theoretical hypothesis model for the adjustment of envy-leadership relationships through the preceding work and the path developed in the literature. On the one hand, this theoretical model can pave the way for empirical work in subsequent studies, and other scholars can test the stimulus–organism–response path in turn based on this paper’s model of ideas (Klein 2011). It clarifies the trajectory of leadership interventions and guiding ideas for envy adjustment (pursuing decision performance or fairness, guiding organizational practice or effectuation), analyses the trajectory of the role of envy in the workplace (single element role of perceptions, attitudes on envy; or mediating, moderating model of perceptions-attitudes-envy), and finally obtains specific adjustment measures. On the other hand, the model creatively points out the role of leadership in the workplace on employee or organizational envy, i.e., it integrates the two sides of envy and leadership while forming a new direction of research under a unified research framework.

Overall, this paper breaks away from the traditional psychological research paradigm of envy and leadership concept analysis. On the one hand, it constructs a two-track model of both, aided by management and econometrics (and even by big data and computer disciplines), and proposes practical ideas and hypothesis paths for subsequent cross-sectional research. On the other hand, the response to leadership adjustment, control, or transformation envy is foreseen in the context of binary relationships and the workplace.

## 2. The Theoretical Basis of the Relationship between Envy and Leadership

### 2.1. Concept Definition

#### 2.1.1. Envy

In the process of analyzing the literature, it is not difficult to find that the English definition of “envy” can describe either of the technical terms “envy” and “jealousy”. Before 2000, scholars analyzed the concept of envy and put forward the concept that when a person both lacks and desires to possess the excellent qualities, achievements, or possessions of others, “envy” is produced, which is accompanied by guilt, self-denial, and inappropriate malice (Salovey and Rodin 1984). “Jealousy” occurs when people fear losing important interpersonal relationships due to competition, accompanied by fear of loss, anxiety, and doubts and anger about betrayal (Smith and Kim 2007). Silver believed that the reason for the mixed use of the two terms is related to the ambiguity of the language (Bedeian 1995), but he pointed out that the most essential difference between the two is that “envy” is a binary relationship, but “jealousy” is a ternary relationship. In addition, the author used an empirical comparison to study the causes and consequences of “envy” and “jealousy” in the article, and concluded that self-esteem, authoritarianism, the attributes of the work environment (competitive rewards, employee autonomy, and leadership considerations), and individual response variables (based on organizational self-esteem, sense of control, and turnover intention) are more relevant to envy than jealousy. That is, the scope of envy is wider than that of jealousy, and it feels more intense for individuals. However, when combing through the literature, the authors found that in recent years, most foreign scholars have not strictly distinguished the two. In addition, since this article discusses the relationship between leadership and jealousy in social workplaces, the definition of jealousy by Salovery and Rodin is that envy is jealousy in the context of social comparison.

Envy has several basic characteristics: low self-esteem, desire, and a subjective sense of injustice (Bedeian 1995). When employees compare themselves with colleagues and focus on the qualities, resources, or abilities they do not possess, they show unpleasant negative emotions. This view is generally recognized and widely cited by the academic community (Zizzo and Oswald 2001). In 1999, Smith classified envy into episodic and dispositional according to the nature of jealousy. Episodic jealousy occurs in response to specific events, and dispositional jealousy refers to the tendency of individuals to feel jealous toward others. In addition, envy can be a positive or negative emotion (Li and Li 2005). Therefore, according to the antecedents and consequences of envy, envy can be divided into benign and malicious; benign envy reflects the admiration of a jealous person and the determination to improve oneself (Lu et al. 2012). Malicious envy refers to a person wishing to take what another person has away from them (Vecchio 2000). Psychology represents envy as a psychological state of indifference, devaluation, rejection, or hostility that people harbor towards those who should be united to compete for certain rights and interests (Tang 2008). It is a manifestation of poor interpersonal relations and a manifestation of human feelings. In this paper, we will always refer to envy in previous studies, specifically as follows: two objects A and B in the same system initially have approximately equal energy fields and the same direction, but when A’s energy field is suddenly larger than B’s energy field if B’s mass is smaller than A’s mass, B’s energy field will not be able to limit the increase of A’s energy field but will be driven by A’s energy field to increase, B’s such This desire of B to be driven up by the energy field of A is called jealousy. This is the theoretical basis of our analysis below.

#### 2.1.2. Leadership

In organizational behavior, the personal traits (character) and decision-making behaviors of leaders are usually the hotspots that scholars pay attention to. In the retrieved literature, it is not difficult to find that a leader’s personality characteristics (e.g., narcissism), leadership behavior style (e.g., disruptive and transformational), leadership–member relationship (LMX), leadership work environment (e.g., corporate culture), etc., are all research perspectives developed by scholars. This is also closely related to the development of leadership theory (Schneider et al. 2005). The traditional leadership theory develops with leadership, injecting values into an organization to arouse resonance in the followers. Group leadership theory involves differences in cultural backgrounds that have significant impacts on employees′ psychology and behavior. In the vertical pairing theory, the “in-circle” and “out-of-circle” relationships formed between leaders and followers have important impacts on organizational behavior. In the transformational leadership theories in recent years, charismatic leaders display their own values and beliefs in non-traditional ways in order to arouse the passion of employees and to influence their behavior. At the same time, the research on destructive leadership has gradually entered the public’s field of vision, and its negative effects on the work attitude and behavior of subordinates and on organizational behavior and performance have attracted widespread attention from scholars and entrepreneurs. Therefore, it is not difficult to find that “innate” leadership has the function of influencing organizational behavior and can resolve jealous activities in the interpersonal field through decision-making behavior (Liang et al. 2019). Based on the above analysis, we find that leadership identity theory, leadership behavior theory, and leadership change theory exist in the workplace and other workplaces. Leaders can influence employees’ behavior and change their minds through some manifestation of these theories. Leadership as a managerial activity is essential for effective management of work and the resolution of psychologically undesirable factors of employee envy at work (Gotsis and Grimani 2016). Therefore, in the following analysis, we focus on broad leadership behaviors rather than specific leadership behaviors.

#### 2.1.3. The Relationship between Jealousy and Leadership

Specifically, the field of research on the relationship between leadership and envy mainly involves the following aspects. First of all, leadership traits include the personality characteristics and personal abilities of a leader, such as whether the leader is considerate (Kim et al. 2013). The level of a leader’s interpersonal processing and business abilities has a significant impact on employees′ organizational citizenship behavior (OCB) and psychological activities (such as jealousy and hatred) (Cropanzano and Mitchell 2005). LMX refers to the quality of the relationship between employees and their direct supervisors. The existing literature proves, through normative and empirical research, that supervisors can adjust employee jealousy by improving the quality of the LMX perceived by employees (Kim et al. 2010). Ethical leadership refers to the leadership behavior that promotes this behavior to followers through two-way communication, strengthening decision making through personal behavior and interpersonal relationships (Veiga et al. 2014). The core meaning is that a leader’s behavior is fair, and this motivates employees through the LMX (Dunn and Schweitzer 2006). On the contrary, unfair and unjust leadership behavior will stimulate jealousy among employees. As a result of this differential treatment, the psychology among employees will gradually change from envy to jealousy, with the final result of bad behavior among employees due to jealousy. Therefore, scholars have concluded that through ethical leadership behavior, employees′ organizational citizenship behavior can be adjusted, and employees′ jealousy or destructive behavior can be alleviated.

In the workplace, a key factor in determining whether or not a manager will be better off has become the improvement of emotional management. The seriousness of internal conflicts caused by the inappropriate handling of jealousy between employees and relations between employees and themselves by some managers is also a notable cause. For managers, the level of leadership and emotional intelligence is often the key sticking point for success in the workplace. In the workplace, everyone plays the role of bearer and facilitator of the corporate culture, and everyone is also the influencer and recipient of corporate values. The level of emotional intelligence and the use of wisdom is the lubricant that determines whether the manager can quickly integrate into the new cultural climate and be accepted and recognized by the organizational culture. Using your wisdom to address poor jealous behavior in the workplace is an essential skill for a good leader.

### 2.2. Theoretical Research

According to the literature analysis, envy and leadership has been a hot research topic in the past ten years, mainly focusing on the antecedents and consequences of envy and the influence of leadership on organizational behavior. Often combined with OCB and counterproductive behavior (CWB). The main theoretical basis involves role theory, social exchange theory, leader–member exchange theory, affective event theory, the theory of planned behavior, and attribution theory.

From the perspective of roles, role theory analyzes and studies a person’s social behavior, including role cognition, learning, and expectations. Kim (Leon and Halbesleben 1981) and others proposed that in leadership positions, people’s moral decision making is driven by their tacit understanding of a leader’s requirements. At the same time, the role’s requirements affect their ethical decision-making process. The social exchange theory reconceptualizes interpersonal communication as a social exchange phenomenon. It believes that the driving force of interpersonal communication is “self-interest”, and that people tend to expand the benefits and reduce the costs in interactions. In a review, (Cropanzano and Mitchell 2005) mentioned that a large number of studies based on social exchange theory proposed that there is a significant direct relationship between LMX and OCB, and that employees with higher-quality leadership–employee relationships have better organizational citizenship performance. LMX theory believes that leaders treat their subordinates differently. Organizational membership often includes a small number of high-quality exchange relationships (between members in the circle) and mostly low-quality exchange relationships (between members outside and within the circle). Leaders establish special relationships with a small number of subordinates. These individuals become insiders and are trusted, receive more attention from a leader, and are more likely to enjoy privileges. Kim (Leon and Halbesleben 1981) and others proposed that employees with low-quality LMX are less likely to have the good support advantages of their colleagues and the resources from relationships, and therefore are more likely to break employees′ sense of balance and produce envy. Based on this, it is proposed that LMX is a precursor to envy, and that hypothesis testing should be performed. The emotional event theory believes that the behavior and performance of employees at work are not determined by attitude and personality to a large extent but are affected by emotional changes at work. Events and conditions in the work environment constitute “emotional events”, which greatly determine mood and emotions. Veiga (Cropanzano and Mitchell 2005) and others used the five basic elements of triggering work, event, evaluation, emotional response, and reaction as a framework to build an extended model that triggers envy events. Based on the theory of planned behavior, Dunn (Silver and Sabini 1978) proposed, in her article, that organizations can influence the moral behavior of employees in a variety of ways. Through the establishment of a model, it was explained how envy at the individual and group levels affects the decisions of individuals to make specific unethical behaviors. In the model, the author considered the mediating influence of envy on attitudes and norms, focusing on the comparison between the individual level and the group level. Leon (Festinger 1954) and others used attribution theory to establish a predictive model of schadenfreude and its impact on working relationships by identifying “how employees perceive the environment”, the “supervisor’s behavior”, and “what happens to colleagues”. “Judgment” and “emotions of the observer” were also considered relevant. Regarding the theoretical research, we have summarized and sorted them out in Table 1.

### 2.3. Empirical Research

#### 2.3.1. Antecedents of Envy

There are many factors that induce envy, mainly involving the following aspects: Envy is produced by unfavorable comparisons. When there is a lack of more objective standards, individuals make social comparisons (Schaubroeck and Lam 2004). Expectations of performance can also affect envy. Schaubroeck and Lam (Duffy and Shaw 2000) found that uncompromising tellers with a high promotion rate are more jealous than uncompromising tellers with low expectations. In addition, to some extent, when a person performs poorly, envy must be stronger (Salovey and Rodin 1984).

#### 2.3.2. Consequences of Envy

Envy can have both positive and negative effects. From a positive perspective, envy can motivate a person to improve performance or to try to improve themselves (Dunn and Schweitzer 2004). On the contrary, when the feeling of envy continues, it also leads to various harmful results, including gloating, attacking someone, and even committing a crime. Envy can not only stimulate the individual to demean and harm the jealous target but can also stimulate the individual to demean and harm a person that has nothing to do with the target (Kim and Glomb 2014). A lot of the literature has proved, through research, that the impact of envy on the operation of an organization is harmful, via moral disengagement and social destruction, and causes harm to outstanding peers (Cohen-Charash and Mueller 2007). In summary, the behaviors caused by envy can be categorized as follows: The first dimension classifies behavior as constructive or destructive (that is, whether the behavior has a positive or negative impact on the organization); the second dimension is based on whether the person who feels jealous includes the goal of said envy in his behavior.

#### 2.3.3. Envy and Leadership

Since leadership is the most powerful resource related to salary increases, better job distribution, or promotions, employees who have a closer working relationship with their supervisors are expected to obtain better support from this relationship (Vecchio 2005). When a leader is not considerate or when followers feel that the quality of their relationship with a leader is low (Castro et al. 2004), envy among peers is more likely to occur. The research results show that leadership can play a role in reducing the emotional burden of employees. In other words, leaders may increase the feelings of envy among some employees because employees feel low-quality LMX. Once employees feel jealous, they often give up their organizational citizenship behavior, and vice versa; this promotes better customer service and employee performance (Schyns and Schilling 2013). In addition, poor leadership ability indicates a tendency to target supervisors′ anti-productive behaviors and damages organizational functions (Hupka 1984).

## 3. The Main Content of the Research on the Relationship between Leadership and Envy

### 3.1. Introduction to the Method

Bibliometrics is a cross-cutting science that uses mathematical and statistical methods to analyze all carriers of knowledge quantitatively. It is a vast body of knowledge that integrates mathematics, statistics, and bibliography and focuses on quantification (Tranfield et al. 2003). The main objects of measurement are the volume of literature (various publications, significant journal articles, and citations), the number of authors (individuals and groups), and vocabulary (various documentary markers, mostly narratives). The essential feature of bibliometrics is that its output must be ‘quantitative’ (Magistretti et al. 2020). Many bibliographic issues are challenging to quantify because of the human factors that affect the flow of information in the literature. In particular, due to the high complexity and instability of the literature system, it is impossible to obtain sufficient and valid information to reveal the macroscopic patterns of the literature. The development of bibliometrics relies on the support of mathematical tools and statistical techniques, and the use of more effective mathematical tools and statistical methods will be an essential direction for its development.

The study of the binary relationship between envy and leadership in the context of the workplace contains a large number of research directions, concepts and connotations (as seen in the previous section), and therefore the grasp of the knowledge structure and literature level of this hot topic is more dependent on the “quantity” and “quality” of the literature. This paper uses the CiteSpace-assisted Histcite citation analysis tool as the main method to extend the bibliometric analysis in the formation of envy and leadership theme studies (Garfield 2009).

Compared to traditional qualitative methods of documentary analysis and commentary, bibliometrics has a broader scope of inquiry and makes it easier to capture good commentary, research (Lucio-Arias and Leydesdorff 2008), and capture the changing history of binary relations. The traditional literature analysis method relies more on the researcher’s familiarity with the topic in the field and the degree of the grasp of the ‘main line of historical commentary’ formed through the researcher’s own subjective or literature review. This subjective qualitative research may overlook many essential documents. However, the bibliometric approach, through objective extraction, extensive data mining, and keyword capture and migration of literature, forms a network of literature analysis, which further realistically shows the intersection of envy and leadership, making the review analysis more profound (Feather and Boeckmann 2007).

In terms of mainstream methods, Tranfield et al. (2003) also proposes the SLR approach to literary analysis, a systematic literature regression (Tranfield et al. 2003). SLR better explains the structure of research in a field and allows analysis of how important literature is reconstructed/transformed, integrated, (short-term) collaborated, sustained, perceived, captured, and acquired in the research system. The strength of the SLR approach is to identify “key scientific contributions”, which provides scholars and practitioners with a basis for understanding the current state of relevant topics and taking the proper steps in future action (Ferro-Díez et al. 2020). It is a replicable, transparent, and auditable methodology. In research, SLR follows a formal process of (1) posing a research question; (2) locating the research; (3) identifying selection and evaluation criteria; (4) data analysis and synthesis; and (5) reporting and discussing the results (Tranfield et al. 2003). Overall, the SLR approach to literary analysis is suitable for parts of a research field where the structure is already largely transparent and has no cross-conflict (Zhang et al. 2021). The researcher can stratify the research using synthesis and comparative evidence based on a straightforward premise of the field and the current state. SLR methods are then used to corroborate the stratified perspectives and construct comprehensive models to reveal the relationships between the dependent and independent variables in the research literature and clarify the strong and weak connections between the stratified studies. However, existing studies on jealousy, leadership, and workplace contexts do not have an upfront comprehensive analysis and comparative evidence and lack a specific focus and essential research questions. In the absence of valid inclusion thresholds and exclusion criteria, this research topic does not explain internal hierarchical correlations using textual evidence, making the SLR relatively inapplicable.

Compared to the mainstream method META (meta-analysis) (Stang 2010), the bibliometric approach is more practical and has a lower threshold for use. Meta-analysis is a statistical analysis of a large body of existing empirical literature. The statistical indicators in the relevant literature are analyzed again using the appropriate statistical formulae so that the actual correlation between two variables can be analyzed based on the statistical significance obtained, for example. It first arose and was used in the field of medicine (Chen et al. 1999). The extensive sample nature of medical papers poses a great deal of difficulty for researchers in tracking the frontiers and guiding researchers to expand the boundaries of their research, which leads to the need for researchers to test the collected research samples in a continuous, empirical perspective for evaluation by others. Therefore, meta-analysis must follow detailed and rigorous research steps. Although meta-analysis can analyze the actual correlation between two variables based on, for example, the statistical significance obtained, it is fragile in the binary exploration of jealousy and leadership (Fischer et al. 2009). For one, the existing analysis of the relationship between envy and leadership has few papers of an empirical nature. It lacks statistical significance and variable analysis between the two, making it difficult for meta-analysis to characterize the underlying data, much less qualitatively separate, to assess the stratification of the research sample. Secondly, there are inherently few papers on leadership, envy, and the crossover between the two, and a lack of continuity in performance, which leads to a lack of contextual identification, sample identification, and especially variables lacking research characteristics (theoretical research structure, research design, and types of effects missing) in the use of meta-analytic methods. In contrast, this paper serves subsequent research scholars well by constructing a two-track hypothetical, theoretical model. It is possible to validate the two-track model proposed in this paper using meta-analysis methods to complete the statistics and measurement of the research data.

The study of envy and leadership in the workplace context is relatively new, and Histcite is much more effective for the analysis of literature that quickly enters unfamiliar territory and achieves generalizability. With mapping analysis, the relationships between different literature in a field can be shown directly graphically. It can quickly help us map the history of a field and locate the critical literature in the field and the most recent important literature. With the support of scientometrics and data visualization, CiteSpace can complement Histcite’s history presentation function (Chen 2006) to present further the structure, patterns, and distribution of scientific knowledge in this field. In summary, the SLR approach is suitable for the re-exploration of mature fields and validating specific hypotheses, evidence, and integrated models but cannot capture historical literature and knowledge structures. Meta-analysis is suitable for the exploration of fields with mature empirical models and stable research structures. The combination of CiteSpace and Histcite allows for precise reproduction of the knowledge graph, free from these limitations. Knowledge mapping can form a knowledge cross-network by reproducing themes and presenting critical citation paths, which is essential for forming envy and leadership binary relationships. The method breaks through the subjective and minor volume limitations of traditional literary analysis.

### 3.2. Literature Search

**Figure 1:**
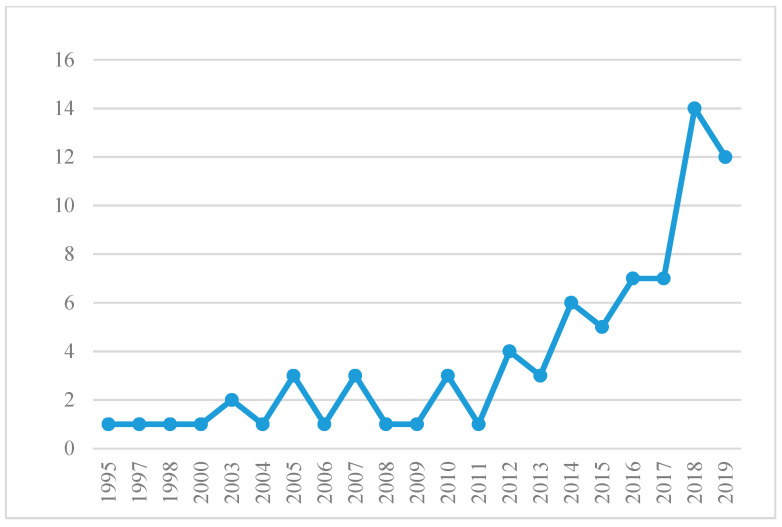
Number of published documents and the trend over the years.

As the international research on organizational behavior is ahead of China, and considering the authority and comprehensiveness of the literature database, this study selected the Web of Science core database as the literature retrieval platform. We combined the explanation and analysis of “Jealous” in the basic theoretical way, using “Envy*”, “Jealou*”, “Lead*” as the search criteria to search all the documents on the platform; the time span was from 1980 to December 2019 on the 21st. The downloaded documents were imported into the citation analysis software Histcite for analysis, and finally 78 documents were recorded, which formed the basis of this quantitative analysis. In terms of time, the overall number of documents was relatively small, rising from one in 1995 to 14 in 2018 (the number in 2019 was 12). This shows that the academic research on this issue has not yet formed a system, but the number of related documents has increased significantly in the past five years, indicating that scholars have gradually realized the importance of the relationship between leadership and envy for the healthy development of organizations. The scale of published literature in each year is shown in Figure 1.

The existing literature (Zhang et al. 2021) data processing paradigm was fully consulted in the data selection, inclusion, cleaning, and exclusion. The value and authority of the articles, the validity of the analysis of the literature, and the potential disciplinary impact and scope of the scholarship were taken into account. We used the Web of Science core databases Science Citation Index Expanded, Social Sciences Citation Index, Arts and Humanities Citation Index, and the Web of Science Citation Index. Conference Proceedings Citation Index-Science, Conference Proceedings Citation Index-Social Science and Humanities, Emerging Sources Citation Index indexes are used as specific search platforms. In the year determination, literature from 2020 was excluded for three main reasons. One, as 2020 is a pandemic year, most academic output countries are in a closed, shutdown state, with relatively few workplaces, envy, and leadership topics, and many psychological scale measures and experimental arguments are at a standstill, making it difficult to use the current year’s literature. Secondly, the literature cited must intersect with the network of the knowledge map. Even though the literature from 2020 is related to the field, it is in a “tail” state, and in the subsequent processing, it is found that it is in a state of disconnection from previous studies (no or few references to previous discussions, out of the original analysis, and challenging to show in the knowledge map). Thirdly, further processing revealed that the concentration of the new year’s literature on the topics above is shallow and hardly falls within the scope of existing studies. Considering the continuity of the citation network and the realistic nodal point for the design of the subject work (the working paper of this paper was completed in January 2021), the 2021 article was not considered for the time being (on the one hand. The cut-off paper needs to be designed to avoid the scattering of research due to the infinite update of the paper. On the other hand, research papers in 2021 are similar to the situation in 2020 and, in the case of the 2020 research cut-off, are far from the center of the knowledge network and lack analytical value). Due to the innovative nature of the topic, this study also points out in its shortcomings and outlook that a new analytical framework for envy-leadership relationships could be constructed in response to the changing workplace climate after the pandemic.

In order to safeguard the research value and analysis effect, this paper formed the inclusion and exclusion criteria for the large sample to the final analysis sample based on the above considerations. Firstly, 218 papers were obtained from the subject term search and imported into Histcite mapping analysis, excluding 41 cut-off articles with broken, uncited relationships. Secondly, the remaining abstracts were incorporated into the CiteSpace software and carefully cleaned according to the subject term hints, and disciplinary categories to remove irrelevant or non-social science papers (e.g., a paper on suppressing jealousy by a particular drug), and a total of 67 articles were excluded. After that, the uncited papers and weakly related papers (lacking a unified dialectic of envy-leadership relationship) were cleaned, and a total of 20 articles were excluded. Then, the text mining software was used to check the main idea and content of the complete text, and 12 invalid papers were eliminated from the selection process. Finally, 78 papers with analytical value, high relevance, and high documentary value were formed.

In an extension of the envy-leadership relationship issue, Pilar (González-Navarro et al. 2018) emphasizes that envy is a common emotion in work environments where competition for resources is intense. As coordinators of management resources, leaders undoubtedly have a significant impact on emotional psychology, such as envy. Despite his use of counterproductive work behaviors (CWB) theory and the construction of envy- leadership research model under the leadership–member relationship (LMX) line of thinking, he still points out in his research outlook that the integration of envy and leadership has been inadequate in academia. This lack of integration is encapsulated in three areas: the lack of a complete and detailed literature base. Most of the existing papers on envy, leadership, and even workplace analysis are single-factor analyses of the literature and lack a fusion perspective. Secondly, there is a lack of analysis of the connotation theory and background history of the relationship between leadership and envy. We can now only observe this phenomenon in the workplace and explain it with particular psychological perspectives and theories. However, theories of analysis under management, sociology, and even other diverse interdisciplinary disciplines are not used, which exacerbates the fragmentation of research. Thirdly, envy and leadership have a dynamic relationship with the times and social changes, and this dynamic relationship needs to be interpreted with a scientific, qualitative, and quantitative approach. In conclusion, the bibliometric approach is the key to integrating envy and leadership research to address the shortcomings mentioned above. Its most tremendous significance lies in its ability to propose analytical models that integrate a more comprehensive range of hypotheses, pathways, and theories. In this way, this paper will examine in detail the changing relationship between envy and leadership by analyzing 78 pieces of literature that are highly intersectional and portray envy-leadership in the workplace relationship.

### 3.3. The Developmental Stage of the Research on the Relationship between Leadership and Envy

Based on all of the documents since research on the relationship between leadership and envy came into being, as well as on visualized analysis according to the time dimension, the research on the relationship between leadership and envy can be divided into three main stages, as outlined below.

#### 3.3.1. Formation Stage: Leadership and Envy in the Story (1995–2007)

**Figure 2:**
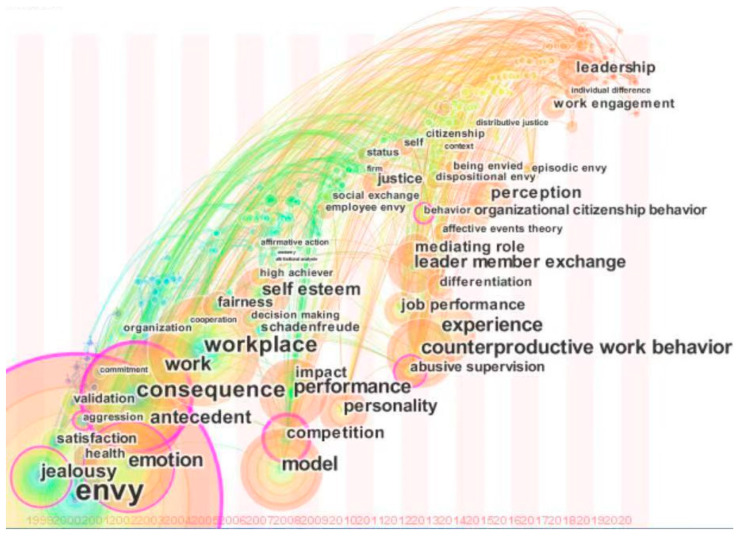
A time zone evolution diagram of the hot topics in the research on the leadership–jealous relationship from 1996 to 2019 (the graph is automatically generated by the software).

**Figure 3:**
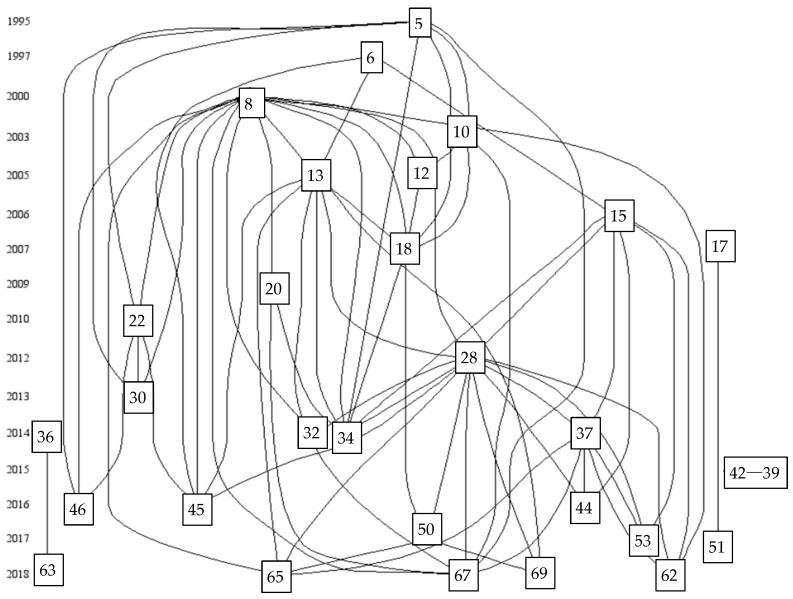
The research context of the relationship between leadership and envy (the graph is automatically generated by the software).

The evolution of the research atlas is shown in Figure 2. The research at this stage has two major characteristics: One is dramatization and the other is ambiguity. Combining Figure 2 and Figure 3, it can be seen that the research on envy originated from the plot in a novel. Through the workplace and environment in the novel, the social construction of envy was put forward, and the changes of envy and its destructive influence on the organization continued to be outlined in “*Othello*”. In essence, the research at this stage did not focus on envy in the work environment (the workplace officially appeared in 2007) but used work and character images that were created in the novel to describe envy. On the contrary, since the concept of envy first appeared in academia, many scholars have searched for suitable terms to express the connotations of envy, and there has been an analysis of the differences between envy and envy. The research was gradually extended to focus on the impacts of envy, including emotions, health, and inner satisfaction, but the core research remained focused on the aggression and other aspects while beginning to form the “outcome theory” of envy research. Finally, with the establishment of a envy research system, academia formally paid attention to the places where envy occurs, including organizations and workplaces. However, in the evolution of this series of studies, envy research has never been specialized, and the research trend is templated as formation–the affected element–the result of the influence. Therefore, in this research path, there are more keywords, new topics are hidden, and the overall trend is blurred.

- Proposal of the Concept

From the quotation diagram of envy research (Figure 2), we can see that there were three influential documents from 1995 to 2005. Document 5 is the first time Bedeian defined the concept of envy—that is, envy is a manifestation of dissatisfaction with the property or attributes of the target. Document 8 proposed and validated nine hypotheses from the emerging literature on negative emotions in the workplace. Document 13 took company executives as the research objects and analyzed the two aspects of feeling jealous, thereby becoming a pioneer in the study of envy, and has a slight color of the combination of envy and leadership.

The important literature and core research keywords from 1995 to 2005 mainly showed two characteristics: First, trying to interpret envy in a theoretical form and to construct a dual research system of envy–leadership, it did not analyze the relationship between envy and leadership in depth, but recognized that envy is a common psychological activity in the workplace; second, certain research methods were proposed, including situational analysis, and case studies, mainly to explore how to apply a psychological research paradigm for the study of envy activities, but quantitative research has not been introduced and the theoretical thinking is still in a fuzzy embryonic state.

- Development of Relationship Research

With attention being paid to the negative effects of envy, academic circles have gradually started paying attention to the driving factors of envy and the effects of jealous activities. The main contents include the production and action mechanism of envy in the body and mind, the vagueness of envy judgment, and the prior elements of envy.

First, there is a driving force for envy. For example, Castro et al. (2004) analyzed the relationship between supervisors and envy, and the research results showed that supervisors can play a role in reducing the emotional burdens of employees. In other words, supervisors may increase the feeling of envy among some employees because of the low-quality LMX that employees feel. Once employees feel jealous, they often abandon their organizational citizenship behavior. On the contrary, it can promote better customer service and employee performance. This document potentially puts forward the relationship between leadership and employee envy, but most scholars at the time explored the causes of envy at a wide level, and did not pay attention to the decisive factors of the jealous activities of leadership. Schaubroeck and Lam (2004) found that employees (behaviors) themselves can also affect envy. That is to say, the greater the expectation, the greater the disappointment. Along with the comparison between one’s own disappointment and expectations, and between one’s own failure and the success of others, envy arises (Silver and Sabini (1978) pointed out, that envy has several inherent characteristics: low self-esteem, desire, and subjective injustice). In the process of exploring the causes of envy, the academic circle gradually paid attention to the key role of leadership in the interpersonal field on envy (2007 and later) and, at this stage, determined the main theories for the study of envy, including social exchange theory, leadership–member exchange theory, and planned behavior theory. Based on these basic theories, an extended model of envy research has gradually been developed, and more leadership perspectives have been incorporated.

Second, there is the effect of envy-linked activities. The direct recipients of jealous behavior are mainly divided into two categories: individuals and organizations. The research focus presents the transition from the individual to the organization. For example, Dunn and Schweitzer (2004) found that envy not only motivates individuals to demean and harm a target, but also motivates individuals to demean and harm goals that have nothing to do with said target. Vecchio (2005) further described the characteristics of the two behaviors caused by envy. The first dimension classifies the behavior as constructive or destructive (that is, whether the behavior has a positive or negative impact on the organization); the second dimension is based on whether the person who feels jealous includes the goals of envy in their behavior (personal). Based on the perspective of individuals and organizations, other scholars have added the harmful consequences of envy, such as a loss of health, a loss of environmental regulation and satisfaction, and undermining organizational stability. However, these conclusions are, first, too broad, failing to elaborate on the effects of envy in a specific situation, and not considering the impacts of subject differences on envy; second, the model is too simple for constructing a universal model.

In summary, the main framework for the study of jealous behavior was basically formed in 2007, so this article defines this stage as the formation stage. Due to its rich story and contextuality, although the research at this stage covers the relationship between leadership and envy, and the envy change mechanism in the interpersonal field, the overall research is too simple; the classic model that was formed gradually became a research paradigm, and subsequent research has found it difficult to innovate. The elaboration of the relationship between leadership and extreme duality needs to be deeply explored.

#### 3.3.2. Development Stage: Leadership and Envy in the Interpersonal Field (2007–2013)

As envy is caused by interpersonal activities, envy frequently occurs in the workplace. Based on this, the research on envy began to shift from focusing on envy in the work environment to focusing on this field, and scholars believe that the production and influence of envy at work is the core part of envy research. The influence of leaders on envy and the mechanism of envy in this field are more general and universal. Explaining the dual relationship between leadership and envy in the interpersonal field is helpful for portraying the overall connotations of envy and provides an effective research path and model for organizations and individuals to reasonably recognize envy.

Early studies on the duality of envy and leadership focused on competition in the workplace; they believed that envy originates from the party that fails to compete in the workplace. However, in fact, even if the competition is separated from the job, there is still a contrast of performance in the work. Even if there is no direct confrontation or conflict of interests between the two parties, due to the existence of leadership, employees compete to improve their expressiveness. This kind of invisible competition has, essentially, no results (which employee is promoted, or which employee has a salary cut), so there is no problem of failure. This kind of competition without judging standards creates a deeper level of envy: all employees establish standards on their own, guided by self-esteem, and form their own jealous feedback mechanism (impact). Once an individual thinks that their expressiveness is not as good as that of others, or thinks that others are secretly closely related to leaders and others, they are likely to experience jealous thoughts. Due to subjectivity, this envy seems difficult to control. For example, in document 8, a “sense of control” was analyzed as a variable of the personal response dimension. However, in workplace activities, leadership still seems to be trying to calm down this jealous idea caused by personal reasons. Most leaders adopt affirmative actions in decision making to maintain fairness as much as possible. For example, Khan, in document 36, found that the stronger an employee’s perception of justice, the less jealous and anti-biological behavior they have. The leadership here seems to have become a balancing method for non-other envy, maintaining a harmonious office environment, and eliminating the pressure of high achievers on others as much as possible. For example, Kim, in document 37, proposed that employees with better performance are vulnerable to other employees′ sabotage behavior, but the team identity formed by a leader can alleviate this adverse effect. At the same time, due to the existence of schadenfreude, once the leadership is out of balance or a gap is broken, the office atmosphere will burst, making it difficult to carry out follow-up work. For example, Leon observed document 39 and found that employees would be pleased with the suffering of others caused by the abuse of supervision by a leader.

With the deepening of research around this situation, scholars have established an empirical research system that begins to discuss how to use leadership correctly and interprets the formulation methods of leadership strategies under different theories and different environments, including social exchange theory (social exchange). Nowadays, the theory is no longer just used to interpret the causes of envy, but also to try to explain the social exchange problem in the office, hoping to optimize the workplace atmosphere. That is, people tend to expand benefits and reduce costs or tend to expand satisfaction and reduce dissatisfaction during interactions. The theory advocates that people should try to avoid competition in conflicts of interest, and that a win–win or multiple wins should be achieved through mutual social exchanges. As the leaders of this exchange, managers must alleviate conflicts, create win–win situations, and maintain a just environment.

In summary, the relationship between leadership and envy is a new direction that combines interpersonal theory and psychology. It has both the management aspects from the human resources discipline and psychology aspects from the social sciences. Management is used to quell psychological gaps and problems, look at envy in the interpersonal field from a management perspective, and build a management–psychological dual model to better portray employee envy (employee envy). Judging from the literature review, the research in this field at this stage is still insufficient, and a complete system has not yet been formed.

#### 3.3.3. Maturity Stage: Multiple Envy and Multiple Leadership (2013–Present)

It can be seen from Figure 3 that, after 2013, research keywords increased, ushering in a new level of research. At this time, the research presents multiple characteristics, and the research on envy is divided. Some scholars believe that envy has a positive side. For example, Duffy believed that envy may produce positive results. It can motivate a person to improve performance or try to improve themselves. Well-intentioned envy reflects the admiration and determination of a jealous person to improve. Kiyoung Lee (Lee and Duffy 2019) also emphasized the relationship between envy and learning; other scholars have considered the new characteristics of envy under the influence of leadership, such as episodic envy and dispositional envy. Under this new way of thinking, scholars have also begun to explore the key elements of the formation of this type of envy, and the core difference from envy under universal conditions.

With the evolution of the concept of envy, leaders′ research on the relationship of envy has also differentiated. In terms of theory, it includes leader–member exchange, counterproductive work behavior, abusive supervision, and affective event theory. On the one hand, scholars still focus on the effect of the control of leadership on bad envy, hoping to use the mediating role to weaken the negative effect of envy. For example, document 63 (Lange et al. 2018) proposed that malicious envy is related to Machiavellian and psychopathic behavior, but the manipulation behavior that affects envy can play an intermediary role. On the other hand, academia has started to raise new questions: Is it poor leadership that leads to envy? For example, the abuse of supervisory power can create panic in the office, as well as small groups of employees, so as to better achieve leadership control, but it also creates negative effects such as envy and invisibly. As well as deliberately assessing and quantifying job performance, the employee group emphasizes competition and neglects relationships, and the office atmosphere tends to have a strong smell of gunpowder, which pushes employees to make progress with a high degree of tension and competition. In terms of models, model elements also tend to be diversified. For example, (Braun et al. 2018) analyzed the performance of followers of narcissistic leaders and believed that they are more likely to respond with higher malicious envy. He built the analysis model which incorporates anti-productive behavior (voluntary, potentially destructive, or harmful behavior that harms colleagues or organizations). Some scholars have incorporated the model elements of the OCB model (employees′ extra helping behavior, directly beneficial to the individual and indirectly beneficial to an organization), using it as an intermediary variable to examine the relationship between moral leadership and workplace envy. Finally, it studies how ethical leaders can influence followers′ participation, organizational identification, and envy through meaningful work. In the last three years, research on differentiation has become popular—that is, the impact of individual differences on envy, leadership, and the relationship between the two.

In summary, the research on the relationship between envy and leadership tends to be diversified, opening up more perspectives. At the same time, we began to actively study the dual problems of envy and leadership, but the overall situation is limited by factors such as corporate characteristics (Thompson et al. 2016), internal organization interactions (Cuddy et al. 2013), leader heterogeneity (Gotsis and Grimani 2016), and industry categories (Leach and Spears 2008). Thus, the research needs to be further explored.

### 3.4. Analysis of Content

#### 3.4.1. Research Topics on the Relationship between Leadership and Envy

We counted the frequency of the subject words appearing in the relevant documents published in all years. The top 100 topic words in the frequency ranking were checked; the meaningless words (causes, analysis, etc.) were removed, identical words (behavior and behavior) were merged, and 55 meaningful topic words were finally retained. According to the main research content of the literature, it was summarized into five themes: The study of the cause and effect of the relationship between leadership and envy, the study of the subject of the relationship between leadership and envy, the study of the subject’s behavioral characteristics, the study of specific behaviors, and the study of the subject’s psychological elements.

According to Table 2, there are 12 key words that appear frequently in the antecedents of the relationship between leadership and envy in the previous studies. The key words (influence, role, moderating role, model, antecedent, predict, etc.) reflect the empirical study of envy and leadership as independent, dependent, mediating and moderating variables, respectively. The emergence of these keywords is indicative of the changing rationale for the analysis of the envy-leadership relationship (see below).

Table 3 shows that perception is the most frequent of the psychological factors in the relationship between leadership and envy, with the theme words “perception”, “recognition”, “attitude”, “understanding” show the psychological, perceptual route of the subject. Among the psychological factors of the subject, narcissism, neuroticism, and sensation are important characteristics that influence the relationship between leadership and envy.

The analysis of the results in Table 4 shows that the workplace, as a specific environmental factor for studying the behavior of leaders and employees in envious relationships, appears with the highest frequency of research. The actors in the relationship between the two reflect the main subjects of study: colleagues (members), workers (followers), leaders (supervisors), collectives (teams), and organizations. From the analysis of the actors, the workplace is the place where envy is triggered, displayed, and carried by leaders, organizations, and workers, so many scholars will focus on the potential mechanisms of environmental influence on envy. When individuals behave in groups, they will undoubtedly expand the scope and influence of envy, so some scholars have begun to explore the variations of colleagues, collectives, followers, and teams in the envy-leadership dichotomy. There are three points here: one, the point-to-point influence, where a colleague who becomes envious transmits this emotion to someone that he or she knows well in the presence of weak leadership interference. The latter’s feedback will determine whether he or she will accept this envy because of the relationship of closeness and trust, and once accepted, this indicates a lousy expansion of envy. Secondly, the influence of the group organization on the group organization, i.e., the idea of envy between different organizations within the company due to competition or other elements, thus creating a confrontation between teams. Thirdly, the team’s influence on the individual, i.e., the creation of the concept of “followership”. In the workplace environment, when the group or groups in which employees are working complain in unison about injustice and create envy, the individual employee will often join them for fear of exclusion. This idea of followership also contributes to the undesirable spread of envy. In summary, existing research has largely adequately considered all the subjects and categories involved in the potential envy–leadership relationship, setting the stage for this paper’s full consideration of the relationship.

Among the theme words under the subject behavior trait study in Table 5, “competition”, “comparison”, “exchange”, “interpersonal”, and “abuse” were the subject behaviors that triggered envy before. “Schadenfreude” and “involvement” focus on the inner activities of the subject when envy occurs. “Rejection”, “mobbing”, “caring”, and “bullying” is more subject behaviors triggered by envy. Table 4 and Table 5 show that the behavior of each type of subject in the envy-leadership relationship is differentiated as the subject of the behavior is identified. From the perspective of the binary relationship, i.e., the leadership’s path to generating and regulating envy, it includes the means of “bullying, replying, being healthy and caring”. This includes positive ways, i.e., appropriate leadership behavior, and negative ways, i.e., inappropriate leadership behavior, which are not purely motivated. In terms of inter-subjective patterns of influence, the first is that in the peer-to-peer transmission of personal envy, the primary means are “gloating” and “interpersonal interaction”. In the case of group-to-group influence, this includes such behaviors as ‘conflict’ and ‘competition.’ In the case of the group’s influence on the individual, it mainly involves “exclusion” and “mobbing”. It can thus be seen that changes in the way envy-leadership relationships are conducted can lead to differences in the performance of each subject’s behavior.

Table 6 shows that the subject terms ‘counterproductive’, ‘ethical’, ‘well-intentioned’, ‘malicious’, and ‘malicious’ under specific behavioral traits reflect scholars’ definitions of the nature of different behaviors. etc. reflect how scholars have defined the nature of different behaviors. Combined with Table 4, it can be seen that the core starting points of the subjects behind the different models and behaviors are not the same. This leads to the behavior of each subject potentially influencing the ultimate scope and attributes of envy. However, competent leaders use positive leadership skills such as “GOOD” and “Ethical” to moderate envy. This ultimately leads them to the right side of “Benign”.

Combined with the reproduction of the theme words, this paper develops an analytical rationale for the dual relationship between envy and leadership. This includes a methodological dimension and a theoretical dimension:

At the methodological level, a network of knowledge literature for analyzing the relationship between the two was formed due to the tandem of subject terms in the envy and leadership theme studies. The relevance of the research is further reinforced by CiteSpace’s use of the principle of scenario and research co-occurrence. Among other things, Thomas Kuhn’s structural theory of scientific revolutions gives CiteSpace a philosophical foundation; the advancement of science is a reciprocal and endless process built on scientific revolutions (Low 2017). This process results in one scientific revolution after another, and people’s understanding embraces new perspectives through scientific revolutions. The importance of the new perspective lies in the ability to provide a more convincing explanation of the object we are observing. The topic of the analysis of the relationship between envy and leadership is clearly in line with this theoretical foundation, i.e., the dynamic envy, leadership connotations, and representational changes that lead to the old and new transformation of scientific cognition. Another principle of CiteSpace is the structural hole theory. Ronald Burt proposes it at the University of Chicago in his study of social networks and social values, and he emphasizes that there is a link between people’s position in a social network and the quality of their views (Haris et al. 2016). The theory of structural holes in social networks can be extended to other networks, especially citation networks. Burt’s structural holes and Kuhn’s paradigm shift are crystallized in CiteSpace. Kuhn’s paradigm is embodied in the clusters that emerge from one time period after another. Burt’s structural hole connects the different clusters. On the other hand, envy and leadership occur more often in the workplace, which is typically a social network of relationships. Social networks externalize internal emotions to connect with different perspectives, expressions, and behaviors, generating entirely new clusters. Histcite works with the principles of big data, text mining, and knowledge networks. The intersection of envy and leadership research is a manifestation of their relationship, their path of action, and this path requires knowledge networks to achieve aggregation. Therefore, the research work in this paper is highly compatible with the methodological principles and can be actively pursued for subsequent analysis.

At a theoretical level, envy, leadership, and the workplace environment constantly adapt to changing times, and the analytical principles on which this is based are constantly changing. In the early workplace environment, leadership had more control over the organization. Theories for the analysis of antecedents of leadership and envy relied heavily on motivational theory, two-factor theory, which emphasized the absolute control of leaders over members of the organization (Braun et al. 2018). Envy at that time, on the other hand, stemmed from the neglect of leadership. The solution is to eradicate envy and its adverse effects by using flexible means of “motivation” or violent means of “command”. This corresponds to the role, status, and personnel change rate theme words in the table “Causes and Consequences”. In the modern workplace environment, where employees are more accessible and subjective, benign envy is likely to generate a positive office climate (Demirtas et al. 2017). His leadership began to emphasize a power shift model to respond to employee jealousy flexibly and appropriately and actively transform non-benign envy. At this time, to consider the influence of employee groups and discern their psychology from the employee’s perspective, the leadership–envy relationship began to apply leadership member exchange theory and affective event theory as the basis for analysis. These theories will be used as a basis for further verification of their support and the logic of their derivation in the various literature, which corresponds to the keywords “moderating role”, “role”, “structure”, and “prediction”.

#### 3.4.2. The Focus of Previous Literature Research and the Relationship between Mutual Citations

In order to sort out the context of the research on the relationship between leadership and envy and to find out its key content, this article used a citation analysis software to draw a context map. Histcite, as an internationally recognized citation analysis tool, chooses to use the local citation score (LSC) as an evaluation indicator of the importance of the literature when drawing.

In Figure 2, we can see the top 30 important documents and their mutual citation relationships in the LCS ranking from 1995 to 2018. Each number represents an article (the software automatically numbers the documents). In addition, the arrow represents the literature citation relationship. For example, document 5, as the source literature, is cited by many subsequent works, so it is connected with many documents. Document 5 is the article “Workplace envy” published by Bedeian in *Organizational Dynamics* in 1995 (Li and Li 2005). The author found that there was very little research on organizational behavior in academia at that time, and the few works that there were rarely involved potentially destructive roles such as workplace envy. Therefore, an exploratory analysis of this widespread emotion was carried out, and guidelines for how to avoid its negative consequences were put forward. In the aforementioned article, Bedeian defined and distinguished the concept of envy, and proposed this: Envy describes a binary relationship involving a jealous person and a jealous target. In addition, envy is not a paradigm of equivalent exchange, but a manifestation of a jealous person’s dissatisfaction with the property or attributes of a jealous target. Regarding malicious and non-malicious envy, the author believed that the tendency of non-malicious envy and the expression of goodwill reflect one’s determination or admiration for others. Malicious envy represents the slander of others. Even though malicious envy is also regarded as a way of motivating one’s own development, it should be condemned. In addition, the author found that the factors that lead to envy are mostly related to personality, an inferiority complex, and belief in the pursuit of excellence, which have an important impact on the establishment of interpersonal relationships and the process of social learning, etc., and proposed from the dual perspective of “leader–employee”. The principles of eliminating envy in the workplace are: Establish a strong team spirit, retain information related to yourself, never show off your achievements and honor, set up a mechanism to alleviate envy within the company, and eliminate pathological envy systems in time. Since then, most of the literature has also carried out research based on this article.

Through analysis of the relevant literature in Figure 2, the developmental context and main content of the research on the relationship between leadership and envy in the workplace can be deduced. We take one of the citation relationships as an example to illustrate the development and inheritance of its research (marked with a solid box in the figure). The source document is document 5, as specified in the previous paragraph. In document 10 (Patient et al. 2003), the authors analyzed plots in novels about envy in the workplace as a case study and explored how envy in the workplace constructs society. On the one hand, Patient put forward the “catalyst” characteristics of envy on the basis of Bedeian’s work, believing that envy can simultaneously stimulate action and sensory changes. On the other hand, it was discovered that envy has the function of reshaping the moral and cultural order of the workplace. In document 12 (Poulson et al. 2005), the author continued Patient’s research method, choosing the content of the chapter “The Tragedy of Othello: The Wasteland of Venice” from the classic Shakespeare novel “*Othello*”. Taking the story of Iago’s dismissal to be promoted, and ultimately causing serious harm to the general’s personal and professional life, as a case study, he studied the destructive emotions in the organizational hierarchy, especially the emotions of envy, anger, and shame. Poulson also studied specific emotions in organizational life and how leaders manage interpersonal relationships in this article. In document 18 (Vidaillet 2007), the author hoped to further develop Lacan’s theory in organizational research on the basis of previous literature. The framework based on Lacan’s theory offers a better understanding of envy in the workplace, while integrating existing research on jealous behavior in organizations. Bedeian once proposed that envy is a binary relationship, while Lacan’s envy theory considers it to be a triangular relationship. It involves a jealous subject, a jealous person, and an observer. At the same time, Lacanism outlined the “dual” role of envy; this theory provided a theoretical basis for later research on the relationship between envy and the “narcissism” of supervisors. In document 50 (Dineen et al. 2017), the authors used a definition of the concept of envy from the previous literature and were inspired by its relationship with personal behavior. In the end, the choice was based on time theory to explain how the envy generated by painful social comparison emotions can be transformed into personal abnormalities (resume fraud) or standardized job search behaviors (working hard). The authors proved, through research, that time variables increase the pressure of job applicants, and further amplify the influence of envy on their deviant job-hunting behaviors. In addition, contrary to the authors’ prediction, market pressure has not only not eased, but has exacerbated the relationship. Dineen et al. explained this phenomenon from a theoretical perspective and discussed possible future research.

From Figure 3, we find that the (Vecchio 2000) circle for literature #8 is the largest and that this literature has the highest citation rate relative to the rest of the literature in the figure, suggesting that this literature is significant in the professional field. Literature #8 examines negative emotions in the workplace, summarizes nine hypotheses from the novel literature on negative emotions in the workplace, and presents nine hypotheses for 167 employed students in bivariate and stratified regression analysis. Literature 13 (Vecchio 2005) and literature 8 are by the same author, and literature 13 is also more prominent in the figure, indicating that the author has a strong track record in this area of research. Literature 13 builds on literature 8 by examining 222 first-level supervisors, and the study concluded that highly Machiavellian people felt the most envy by their colleagues. Document 18 (Vidaillet 2007) summarizes Rebort’s research site and context by introducing Lacanian theory to the study of workplace envy, and the analysis concludes that Klein’s approach emphasizes the differences between envy and jealousy and the position of one person’s dependence on the other. In contrast, Lacan’s theory outlines the ‘dual’ role, the symmetrical position of jealousy and envy, and the alienation of the envied person. Literature 50 (Leheta et al. 2016) also examines changes in envy in the workplace, but from the job seeker’s perspective rather than the leader, which is different from the cited literature 18. The literature is different from the cited 18. The authors’ study found that: we theorize that as job searches progress across time or discrete events, temporal-based pressure increases via perceptions that situations are less changeable or more critical, propelling envious job seekers toward deviant rather than normative search behavior. Literature 65 (Lee et al. 2018) developed and tested a theoretical framework describing the dual affective and motivational experiences that arise from feelings of being envied in the workplace. We can find in this literature that they have in common the study of envy behavior in the workplace, with differences being studied from different perspectives such as leaders, job seekers, and general employees. The basis of all this research is based on literature 8 for the study, which proves the importance and the cited relationship of literature 8 in Figure 2.

Analyzing the remaining literature in Figure 3 through the same method, it was found that a research system of leadership and envy has basically been formed. Scholars have sorted out the causes and consequences of the relationship between the two and have used normative and empirical research methods to conduct theoretical and empirical analysis. However, after a careful review of all of the literature, it was found that in recent years, there has not been a new research perspective in this field.

## 4. Core Word Mining and Model Construction

**Figure 4:**
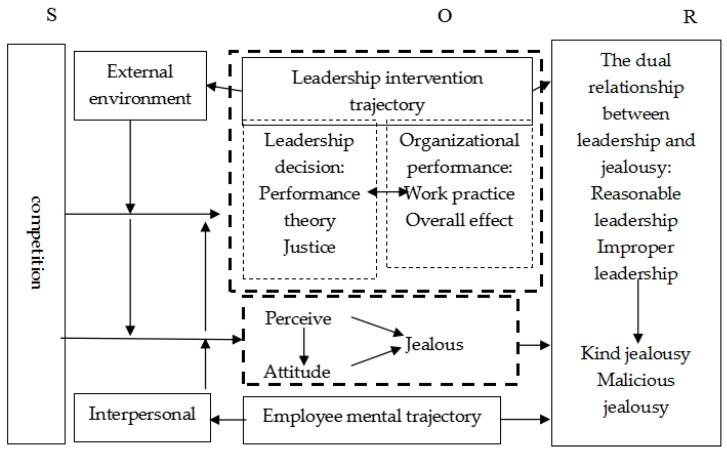
The dual-track relationship model of leadership and envy.

CiteSpace software can realize the visual analysis of a keyword clustering network, so as to obtain the hotspots and focuses of research in this field, that is, the knowledge base. Creating a map of scientific knowledge is one of the important methods of bibliometric analysis. Therefore, this article used CiteSpace software to analyze the keywords in all documents and their citations, and the results are as follows: combined with Figure 4 and Table 7, it can be seen that the research on the relationship between envy and leadership is obviously clustered, focusing on the formation of six sub-research areas of workplace discrimination, influencing performance, leadership behavior, competitive factors, psychological factors, and employee behavior. From the perspective of the logical relationship, the research on envy and leadership originates from a competitive problem (i.e., the competitive relationship described above). On the one hand, envy is caused by environmental factors, including social preferences. On the other hand, it is determined by human factors, including wage differences and ability-matching issues. With the increase in competition, environmental and human discrimination have gradually formed. Environmental discrimination includes competitive issues, such as gender selection, same-sex competition, and racial discrimination. Such problems are mainly caused by a narrow view and misguided social cognition, leading to unnecessary competition and bad relationships. The latter is caused by the fairness of the organization and the aggressiveness of individuals, and the competition is dominated by human factors. As discrimination in the workplace increases, deviations in employee behavior are caused. Employees emphasize the self, and their abilities, character, and willingness are severely divided, resulting in tendentious envy and counter-productive behaviors such as lying and theft. The widening gap between employees and the organization has led to the gradual intervention of leadership, such as avoiding kinship, creating a fair distribution environment, and emphasizing performance first. Additionally, eliminating the problem of victimization in the workplace and striving to avoid overt and visible envy from undermining the office atmosphere are goals. However, leadership can be good or bad. Some managers do not consider the actual situation and blindly rank based on performance. They create a competitive environment, which, in turn, has impacts on organizational and personal performance. Employee friendships are further screened, and organizational performance and work practices determine the final behavioral consequences. With the resurgence of individual competition, people outside the center of things produce complex psychological changes, including sympathy and schadenfreude.

The compound evolution of the relationship between envy and leadership has gradually enriched the relevant research system. Based on the analysis of existing research content and research methods, this article proposes a dual-track model of the dual relationship between envy and leadership under the stimulus–body–response (S–O–R) paradigm, as shown in the Figure 4:

In the interpersonal environment, the essence of any activity is a kind of competition (cooperation can be regarded as the unity of individuals to fight against other organizations or individuals). Whether they are employees or other actors, they often start to act subjectively or passively when they participate in interpersonal activities because they receive competition messages (so competition is the main stimulus).

Relevant studies have shown that in situations with rich external preferences, social subjects with a high degree of involvement involuntarily engage in jealous or repulsive activities. Therefore, it can be speculated that the external environment, including racial prejudice and occupational discrimination, have a moderating effect on the envy–leadership binary relationship. In the same way, internal concepts also guide the direction of the binary relationship and bring about adjustment. Subjects with high standards, high self-esteem, and independent personalities are more likely to have envy, while those with low motivation are the opposite. Under normal circumstances, it is difficult for leadership to break through employees′ psychological defenses and to have a decisive effect on an individual’s psychological standards (inner, man-made), but new standards can ultimately be fed back to the individual’s autonomous standards (body 2) through the intervention of leadership (body 1). In the process of leadership development, the external environment is first screened, and the unfavorable environment that the organization needs to eliminate is judged before starting actions. For example, the discriminatory environment must give way to a fair atmosphere, and the ability mismatched word-of-mouth environment must give way to positive performance standards. Finally, this is reflected in the leadership’s work summary. In the trajectory of employees′ psychological effects, they generally first produce perceptual evaluations of themselves, feed back to their attitudes toward people and things, and act together with perceptions in their jealous behaviors. This process is also subject to the intervention and roles of leaders. In response to the dual-track trend of leadership and envy, an effective response can finally be formed: good leadership can eliminate bad envy and even turn it into positive envy. Inferior management leads to a lack of benign envy, and even malicious envy. In fact, it remains to be clarified by the academic community whether malicious envy is formed because of wrong management or the persistence of malicious envy.

In summary, the dual study of envy and leadership still needs to be further explored, and the duality of envy and leadership will become a new research hotspot. In addition, in terms of research methods, empirical data based on organizations are more reliable in research and more in line with the analysis requirements of the big data era, and the conclusions drawn will be more targeted and effective.

## 5. Conclusions, Shortcomings and Outlook

Overall, this study better fulfills the research objectives presented in the previous section.

At the conceptual level: (1) We elucidated the difference between envy and jealousy. We found that envy is more associated with the environment and more likely to be found in social and workplace settings through specific theoretical analysis and literature exploration. Moreover, the concept of envy has both positive and negative characteristics in its attributes, which means that envy can be regulated and controlled. In the social context, leadership is used more as an emotional tool. (2) In the concept of leadership, we sorted out the patterns of leadership activities and their work intellectualization in various stages, such as “deliberately causing competition among employees to expand corporate effectiveness”. (3) In the interaction between the concepts of leadership and envy, we also discovered the antecedents, factors, subjects, and nature of leadership influence on envy. We emphasize that intelligent leadership, emotional leadership (fully considered from the staff’s point of view), can drive the joyous work of employees. This kind of leadership can circumvent employee conflicts in the workplace, rather than expanding or disregard them.

At the theoretical level, we combed through the history of changes and theoretical innovations in envy and leadership at the theoretical level. In the earliest studies, envy was considered undesirable behavior, and it had no exploitable value in the workplace. Therefore, leadership had to kill envy as soon as it was detected in the team. This is the “envy elimination concept” based on value theory. In the wake of stories such as “*Othello*”, scholars have developed the doctrine of interpersonal relationships, emphasizing that some envy is justified and positive. It can motivate employees to improve, regulate their behavior and work hard to find jobs. Employees’ envy makes them afraid of falling behind. Therefore, the intelligence of leadership at this time is that it can lead to envy to push employees to improve. Modern society has been enriched by the research on leadership member exchange theory and emotional event theory, which emphasizes that leadership and envy should be flexible. Flexibility implies emotional intelligence and social intelligence: not interfering too much but allowing the workplace environment to develop freely.

At the relational level, we also work with goals one by one. We emphasize that envy and leadership are not a single path to control, nor simply linear relationships. Their various sub-concepts have long been intricately intertwined, so it is inappropriate to examine the topic with a single theory or perspective.

The greatest goal and value of our research is to propose a new hypothetical model of the dual-track theory. This model contains most of the previous theoretical paths and forms an analytical path for the general relationship between envy and leadership one by one. It can be used as a basis for subsequent theoretical analysis and to support later hypotheses on the relationship between the two. Most importantly, it is proposed to provide a paradigm for studying changeable wisdom in the workplace (how to use leadership well and how to control or play envy).

In the 21st century, the pressure in the workplace has gradually increased, but the construction of the leadership function under the scope of management tends to be homogenized. Although the Theory of Change and the Theory of Transposition have received much academic recognition, it is undeniable that the “liberalized” management pursued by Change is difficult to summarize in a precise scientific theory but is more a combination of management experience and wisdom. It is more of a combination of management experience and wisdom, which challenges leadership to control the business. In the modern social environment and workplace climate, employees’ sense of autonomy and initiative is greatly enhanced. Many employees will strengthen their sense of protection for the sake of their autonomous interests, especially the post-90s, as they are called, will fight against their leaders for their inner pleasure and spiritual pursuit, which means that Maslow’s hierarchy of needs seems to be an inverted triangle in the new workplace climate, where spiritual pursuits are most important. In this situation, the emergence of the workplace envy mentality becomes commonplace. Some envies are positive in that a group or employee will strive to be ‘good’, to do their job and help others without compromising the interests of others, in the hope of gaining the ‘approval’ of the leader. This kind of envy leads to increased business effectiveness. Destructive jealousy can be detrimental to the interests of the company and the interests of others. In the case of unjustified jealousy, employees compete viciously with each other to the detriment of each other’s work and interests, leading to an internal drain on the company’s resources. Therefore, how to reconcile leadership and envy has become a new topic in contemporary business management. However, in terms of traditional research paradigms, the analysis of this topic is based on psychological paradigms and is primarily purely qualitative. In order to make the transition from qualitative to quantitative research, it is necessary to construct and generalize a complete dual-track model of leadership envy based on extensive literature analysis and then lay the theoretical foundation for the empirical research.

As a result, this paper constructs a paradigm for exploring the envied leadership relationship under bibliometric analysis using the CiteSpace-assisted Histcite method to address the above scientific questions. In the study, the research variations of envy and leadership are sorted out. The two-track model of the envy-leadership relationship in the workplace context is developed by combining the theoretical foundations of management and librarianship and clarifying the path of leadership regulation of envy based on the changes in their relationship. This model is derived from analyzing the envy thoroughly- leadership relationship considering the triggering mechanism of competition in workplace activities and clarifying the competition path on the generation of envy in the workplace. Secondly, the feedback of the external environment and internal, interpersonal elements in the competitive relationship is explored to uncover the decision making and performance appraisal generated by the leader after receiving messages from the external environment and internal interpersonal. Through the leader’s intervention trajectory, perceived attitudes and envy continue to adjust in the employee’s psychological trajectory, eventually transforming into benign envy with the role of positive elements. Thus, in terms of response representations, reasonable leaders can transform malicious jealousy into benign envy and maintain the presence of benign envy. Inappropriate leadership, on the other hand, inhibits good envy and expands the presence of malicious jealousy.

This paper breaks through the traditional paradigm of psychological and documentary analysis and realizes an exploration of the envy-leadership relationship in the context of management, documentary, and graphical sentiment. Using big data and text mining tools to clarify the envy-leadership relationship achieves an innovative breakthrough in methodology, thinking, and theory. However, what is lacking is that this paper also lacks quantitative means of research and does not apply the two-track model directly or give preliminary experimental proof. In addition, the change in workplace climate after the pandemic is also an issue that cannot be considered in this paper for the time being. Subsequent research can be conducted from quantitative analysis and post-pandemic academic discussion to enrich the academic significance of this topic further.

Managers and employees in the workplace are at the heart of productivity, and by resolving conflicts between those involved and by mobilizing the intelligence of all those involved, productivity can be improved. The application of double regulation theory to workplace management practice is a further development of management theory. At the same time, stimulating the intellectual potential of managers and employees, improving the emotional intelligence of those involved and transforming jealous behavior into envy will lead to more efficient resolution of conflicts at work and improve the guarantee for efficient work.

## Figures and Tables

**Table 1:** Analysis of the application of leadership theory.

Types of Theory	Related Literature
Role Theory	(Leon and Halbesleben 1981)
Social Exchange Theory	(Cropanzano and Mitchell 2005)
LMX	(Leon and Halbesleben 1981)
Affective Event Theory	(Cropanzano and Mitchell 2005)
Theory of Planned Behavior	(Silver and Sabini 1978)
Attribution theory	(Festinger 1954)

**Table 2:** Frequency and categories of antecedent and consequential research topic terms in the relationship between leadership and envy.

Cause and Effect	
Effect	13
Moderated	8
Role	6
Model	5
Gender	5
Antecedents	4
Woman	3
Construction	2
Status	2
Turnover	2
LMX	2
Predict	2

**Table 3:** Frequency and category of subject terms in the study of psychological factors in the relationship between leadership and envy.

Psychological Factor	
Perceived	6
Understanding	3
Attitudes	2
Feeling	2
Narcissism	2
Neuroticism	2
Identification	2

**Table 4:** Frequency and categories of subject research theme terms for leadership and envy relationships.

Line as the Main Body	
Workplace	25
Employee	13
Organization	8
Leader	7
Supervisor	4
Member	4
Coworker	4
Group	3
Follower	2
Others	2
Team	2

**Table 5:** Frequency and categories of subjective behavioral studies on the relationship between leadership and envy.

Subjective Behavior	
Behavior	13
Abuse	6
Comparison	5
Schadenfreude	4
Interpersonal	4
Performance	4
Competiton	4
Response	4
Engagement	3
Exchange	3
Health	3
Justice	3
Ostracism	3
Mobbing	3
Bullying	2
Conflict	2
Care	2

**Table 6:** Frequency and categories of topic terms in the study of the behavioral nature of the relationship between leadership and envy.

Nature of Behavior	
Counterproductive	7
Malicious	5
Temporal	2
Benign	2
Deviant	2
Ethical	2
Good	2
Bad	2

**Table 7:** The main clustering of documents based on the Loglikelihood ratio (LLR) algorithm.

Cluster Number	Quantity	Homogeneity	LLR Log-Likelihood Rated Label Words
#0 Workplace discrimination	54	0.882	Sexual selection; intrasexual competition; aggression; body build; racial discrimination; organizational injustice
#1 Affect performance	33	0.773	Consequence; hidden profile; judgment; friendship; performance work practice; organizational performance
#2 Leadership behavior	32	0.774	Leadership; collective identity; relative; workplace victimization; task performance; uncertainty management; distributive justice
#3 Competitive factors	32	0.893	Social preference; wage inequality; competition; wage dynamics; ability
#4 Psychological factors	28	0.88	High achiever; sympathy; resentment; schadenfreude; preference; discrete emotion;
#5 Employee behavior	24	0.816	Self; differentiation; dispositional envy; deviant behavior; lying; employee theft; counterproductive work behavior

## Data Availability

Not applicable.
